# A pseudoproxy emulation of the PAGES 2k database using a hierarchy of proxy system models

**DOI:** 10.1038/s41597-023-02489-1

**Published:** 2023-09-14

**Authors:** Feng Zhu, Julien Emile-Geay, Kevin J. Anchukaitis, Nicholas P. McKay, Samantha Stevenson, Zilu Meng

**Affiliations:** 1 Climate and Global Dynamics Laboratory, National Center for Atmospheric Research, Boulder, CO USA; 2 Department of Earth Sciences, University of Southern California, Los Angeles, CA USA; 3 Laboratory of Tree-Ring Research, University of Arizona, Tucson, AZ USA; 4 School of Geography, Development, and Environment, University of Arizona, Tucson, AZ USA; 5 School of Earth and Sustainability, Northern Arizona University, Flagstaff, AZ USA; 6 Bren School of Environmental Science and Management, University of California, Santa Barbara, Santa Barbara, CA USA; 7 Department of Atmospheric Sciences, University of Washington, Seattle, WA USA

**Keywords:** Palaeoclimate, Palaeoceanography

## Abstract

Paleoclimate reconstructions are now integral to climate assessments, yet the consequences of using different methodologies and proxy data require rigorous benchmarking. Pseudoproxy experiments (PPEs) provide a tractable and transparent test bed for evaluating climate reconstruction methods and their sensitivity to aspects of real-world proxy networks. Here we develop a dataset that leverages proxy system models (PSMs) for this purpose, which emulates the essential physical, chemical, biological, and geological processes that translate climate signals into proxy records, making these synthetic proxies more relevant to the real world. We apply a suite of PSMs to emulate the widely-used PAGES 2k dataset, including realistic spatiotemporal sampling and error structure. A hierarchical approach allows us to produce many variants of this base dataset, isolating the impact of sampling bias in time and space, representation error, sampling error, and other assumptions. Combining these various experiments produces a rich dataset (“pseudoPAGES2k”) for many applications. As an illustration, we show how to conduct a PPE with this dataset based on emerging climate field reconstruction techniques.

## Background & Summary

Reconstructions of past climate have become integral to climate assessments^1^. Such reconstructions employ a wide variety of mathematical techniques, ranging from purely statistical^2^ to data assimilation techniques that fuse observations and model output^3–20^. To establish their relative merits, these reconstructions must be benchmarked against reference datasets. This is routinely done on subsets of the instrumental period using cross-validation, but such efforts tend to underestimate the true spread of reconstructions in the pre-instrumental era^21^, indicative of overfitting.

While pre-instrumental intercomparisons of reconstruction methods have occasionally been carried out with real-world proxy observations^22,23^, such efforts are fundamentally limited by the lack of a true benchmark: pre-instrumental climates were not, by definition, observed directly, so these intercomparisons can only inform on convergence or divergence, but cannot provide any metric of their closeness to the true climate.

To sidestep this hurdle, pseudoproxy experiments (PPEs) have long been used as a laboratory to benchmark climate reconstruction methods. The heart of PPEs is to start from the output of long integrations of a global climate model and to apply mathematical transformations to this output to mimic the processes whereby paleoclimate proxies register these climate variations in space and time^24^. Because the original climate is specified, and sampled perfectly in space and time, the ability of a reconstruction to recover this climate is known. Moreover, as the generating process of these “pseudoproxies” is specified, it can be manipulated to yield insights into the sources of uncertainty contributing to reconstruction error. While simple PPE designs are informative, the more realistic the target climate and pseudoproxy generation process, the more relevant this benchmark becomes, so there is considerable potential in this avenue of research^16,25–31^.

Initial work used the simplistic assumption that paleotemperature proxies were a linear superposition of local temperature and Gaussian (white) noise, sampled uniformly in time^32–34^. Over time, more realistic pseudoproxy constructions were developed, involving other climate variables, more elaborate noise models, realistic spatiotemporal sampling, and noise levels approximating real proxy networks^16,28,35,36^. Recent work^29^ has leveraged more realistic proxy system modeling (PSM) frameworks^37–42^ to capture the essential physical, chemical, biological and geological processes that translate climate signals into the paleoclimate records that form the basis of climate reconstruction efforts (e.g. ref. ^43^). However, such models have yet to gain widespread use, so even recent efforts have sometimes employed a simplistic “temperature + noise” pseudoproxy design^16,29,44^.

**Fig. 1:**
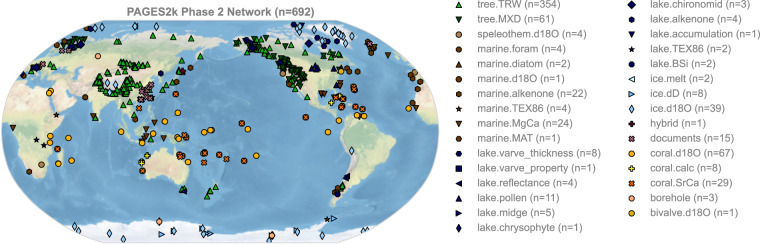
The PAGES 2k Phase 2 network^43^.

The PAGES 2k Phase 2 global multi-proxy database (Fig. 1) has been widely used for studies of Common Era climate since its release^43^. It has played a central role for investigating the multi-decadal and longer-term surface temperature variability^45,46^ and the spatiotemporal temperature patterns of various climatic epochs^23,47^ over the Common Era. In addition, it has served as the principal data source for the latest version 2.1 of the Last Millennium Reanalysis (LMR) products^48^, and has become a common network template for pseudoproxy studies^31,49^. However, these PPE related studies used only a partial network and employed a simplistic “temperature + noise” design, and a systematic pseudoproxy emulation of the PAGES 2k network has yet to be produced. The PAGES 2k Phase 2 has a number of known biases that present challenge to global annual mean temperature reconstruction^50^, which need to be rigorously evaluated.

Here, we do so by generating a pseudoproxy dataset that: (i) emulates the majority of the PAGES 2k Phase 2 network^43^, (ii) employs a more realistic data-generating mechanism with proxy system models (PSMs) and isotope-enabled climate model simulations, and (iii) explicitly separates sensor, archive, and observational effects. By combining various pseudoproxy designs, noise levels, and spatiotemporal sampling scenarios, we generate many digital avatars of the PAGES 2k network, supporting the evaluation of climate reconstruction methods in a wide variety of settings. To illustrate the use of this dataset, we show its application to a suite of climate field reconstructions^10,48,51^.

## Methods

### Reference climate

Our base climate utilizes the “iCESM1” last millennium simulation (iCESM-LM hereafter) generated by the isotope-enabled Community Earth System Model (iCESM)^52^. As an addition to the standard CESM, iCESM simulates the isotopic water fluxes transported between its five major isotope-enabled components, including the atmosphere model iCAM, the land model iCLM, the ocean model iPOP, the sea ice model iCICE, and the river runoff model iRTM. The atmosphere model iCAM tracks water tracers and isotopes in all phases through processes such as surface fluxes, boundary layer mixing, cloud physics, convection, and advection, and simulates precipitation *δ*^18^O variability with high fidelity^53^. The land model iCLM considers the water vapor flux and isotope fractionation in vegetated land surfaces^54^. Main processes include water isotope exchanges among soil, spaces under and above canopy, and leaves. The land and vegetation types and amount of canopy use a modern climatological mean with a constant seasonal cycle^55^. The ocean model iPOP transports water isotopes passively through resolved flow and parameterized turbulence, and the simulated seawater *δ*^18^O is validated under present-day climate conditions^56^. The sea ice model iCICE simulates the sinks of the isotopic water mass through melting and sublimation processes, and the sources through snowfall, sea ice growth, and vapor condensation^52^. All components coupled together provide a plausible simulation of the water isotope fields.

The iCESM-LM simulation applies the transient external forcings following the same setup for the CESM Last Millennium Ensemble (CESM-LME)^57^. The solar forcing comes from the total solar irradiance reconstruction by Vieira *et al*.^58^ patched with spectral variations from Schmidt *et al*.^59^. The last millennium volcanic forcing is based on the ice core-based index by Gao *et al*.^60^, while for the historical period, an eruption dataset by Ammann *et al*.^61^ is adopted. The greenhouse gas forcing, namely the concentrations of the main long-lasting greenhouse gases (i.e., CO2, CH4, N2O), are derived from Antarctic ice core analyses by Schmidt *et al*.^59^. For the land use and land cover boundary conditions, the reconstruction by Pongratz *et al*.^62^ and that by Hurtt *et al*.^63^ are merged together to yield a consistent land use change. The orbital forcing is computed in the model based on Berger *et al*.^64^. The ozone forcing comes from the Whole Atmosphere Community Climate Model (WACCM) and the prescribed aerosol forcing are applied only over the historical period. For more details, please refer to CESM-LME^57^.

### Proxy network

**Fig. 2:**
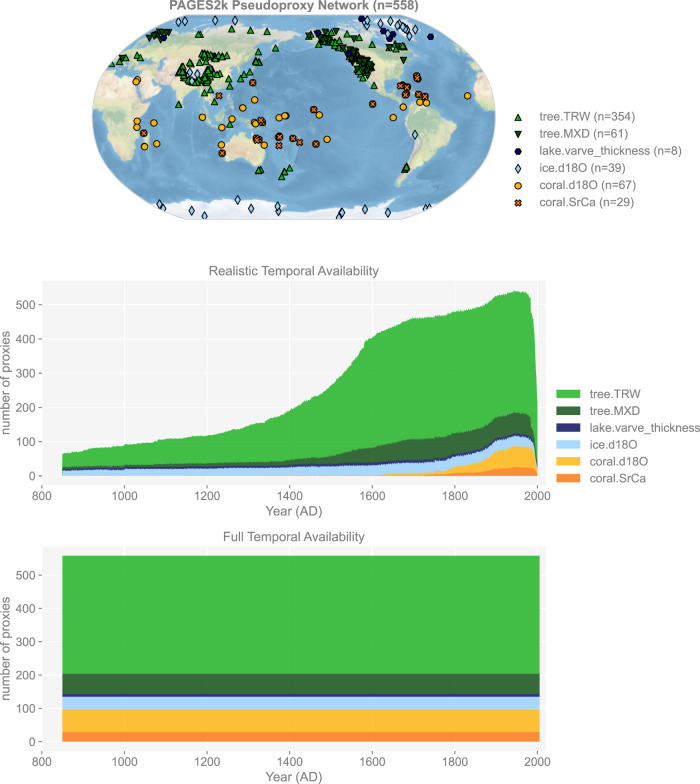
The spatiotemporal availability of the PAGES 2k pseudoproxy network with realistic and full temporal availability.

Figure 1 shows the PAGES 2k Phase 2 Network^43^. It consists of 692 records from 648 globally distributed sites, archived in trees, corals and sclerosponges, marine sediments, lake sediments, glacier ice, documentary sources, speleothems, boreholes, bivalves, and hybrid records. Each archive includes single or multiple observation types, among which tree ring width (TRW), maximum latewood density (MXD), coral and sclerosponge *δ*^18^O and Sr/Ca, lake varve thickness, and ice core *δ*^18^O are essential to Common Era temperature reconstructions (e.g., LMR) and their PSMs have been developed by recent efforts already^38^. We therefore focus on these proxy types, and generate their emulations to form our pseudoproxy network (Fig. 2). For proxy sites located within the same model grid cell, the input climate signals are the same, while the generation mechanisms vary according to their proxy types.

### Proxy system modeling

Following the proxy system modeling framework^37^, we build our pseudoproxy network based on the iCESM output, leveraging the PSMs from the PRYSM toolbox^38^ and the CFR codebase^65^. The concept of PSMs encompasses both geophysical/chemical/biological process-based models, as well as statistical models; both can be either linear or nonlinear. In this study, both categories of PSMs have been adopted, depending on availability. A given PSM can only be applied if its inputs are within the scope of the available climate variables. In addition, the more complex the PSM, the more parameters it contains, and these parameters must generally be fitted to modern observations, lest they introduce additional sources of uncertainty.

As in all modeling endeavors, the choice of PSM is therefore a trade-off between “sins of omission” (excessive simplicity) and “sins of commission” (excessive complexity). The present dataset used the most complex PSMs where justified by scientific understanding and available data. When these conditions were not met, simpler PSMs were selected to avoid sins of commissions or logistical hurdles (e.g. model fields available at too coarse a resolution).

Statistical PSMs, although highly idealized, are still based on scientific understanding of the geophysical/chemical/biological processes leading to the transduction of climate signals into proxy archives. As shown in Tardif *et al*.^48^, even linear, statistical PSMs for tree-ring width that include bivariate and seasonal dependence can yield vastly more realistic results than the traditional fitting to annual temperature.

#### Forward modeling of tree ring width (TRW)

Tree-ring width (TRW) is a major observation source to investigate the Common Era climate. In the PAGES 2k database, TRW represents the largest network with 354 records, most of which are located in the Northern Hemisphere. Depending on the location and species, TRW chronologies may record not only temperature variations but also moisture conditions, although the climatic signals can be modulated by biological memory effects^49,50,66–72^. The relationship between TRW and the environmental variables is thus complex, and TRW PSMs with various complexity levels have been developed since 2000, including TREERING2000^73^, Vaganov-Shashkin (VS)^74^ and its simplified version VS-Lite^75–77^, MAIDEN (Modeling and Analysis In DENdroecology)^78,79^, and even the land model ORCHIDEE (ORganizing Carbon and Hydrology In Dynamic EcosystEms)^80^.

This work used VS-Lite developed by Tolwinski-Ward *et al*.^75–77^ to generate our pseudo-TRW network because of its overall skill^79,81^, simplicity, and capacity to be widely applied to the PAGES 2k sites. VS-Lite takes monthly temperature and precipitation signals as input, and emulates a threshold-dependent tree-ring monthly growth response to the climate with piece-wise linear growth response functions (Eq. (1)) determined by four parameters: the lower and upper thresholds for temperature and soil moisture, respectively:

1 $${g}_{V}(V)=\left\{\begin{array}{lcl}0 & {\rm{if}} & V\le {V}_{1}\\ (V-{V}_{1})/({V}_{2}-{V}_{1}) & {\rm{if}} & {V}_{1}\le V\le {V}_{2}\\ 1 & {\rm{if}} & {V}_{2}\le V\end{array}\right.,$$

where *V* represents *T* (temperature) or *M* (moisture). The overall growth response is then the minimum of these two response functions modulated by the insolation-based growth response (*g*_*E*_) (Eq. (2)), which is determined by the latitude of the site, and the final output TRW is the standardized series of the annual integration of the monthly growths, with an error term added on (Eq. (3)):

2 $$g={g}_{E}\ast \min \{{g}_{T},{g}_{M}\},$$

3 $${\rm{TRW}}={\rm{standardize}}\left({\int }_{{t}_{s}}^{{t}_{e}}gdt\right)+\zeta ,$$

where *t* represents time in month, *t*_*s*_ and *t*_*e*_ denote the window for the integration, and *ζ* is a pink noise term (i.e. a stochastic process with a spectral density $S(f)\propto {f}^{-\beta }$, with *β* a positive constant). Setting *t*_*s*_ < 0 (a specific month in the previous year) can help mimic the biological memory effect or other unaccounted for sources of low frequency variability in TRW^66,69,72,82–85^. Following ref. ^75^, we set *t*_*s*_ = −4 and *t*_*s*_ = 12, which represents an integration window from September of the previous year to December of the current year for the Northern Hemisphere, and from March of the current year to June of the next year for the Southern Hemisphere. The pink noise term is added to further mimic other non-climatic processes such as the detrending process of TRW records, following the formulation of colored noise proposed in ref. ^86^ with tuned spectral scaling slope^87,88^ *β* = 2 and SNR = 1 (signal-to-noise ratio defined in standard deviation^24,26,89^). Without this term, the scaling slope of the simulated TRWs will be significantly flatter than that observed in the real records, especially for the low-frequency band^40^. The need for this can be viewed in two ways: on one hand, it suggests that tree-ring width records in the PAGES2k database contain more low-frequency than expected from the climate signal and simple persistence structure present in VS-Lite alone, perhaps due to data processing (detrending and standardization) or unaccounted for biological or ecological processes. Alternatively, this can be seen as a result of a “sin of omission” in VS-Lite and an incomplete mimic of the full range of biological processes important for the autocorrelation structure of temperature-sensitive tree-ring series.

**Fig. 3:**
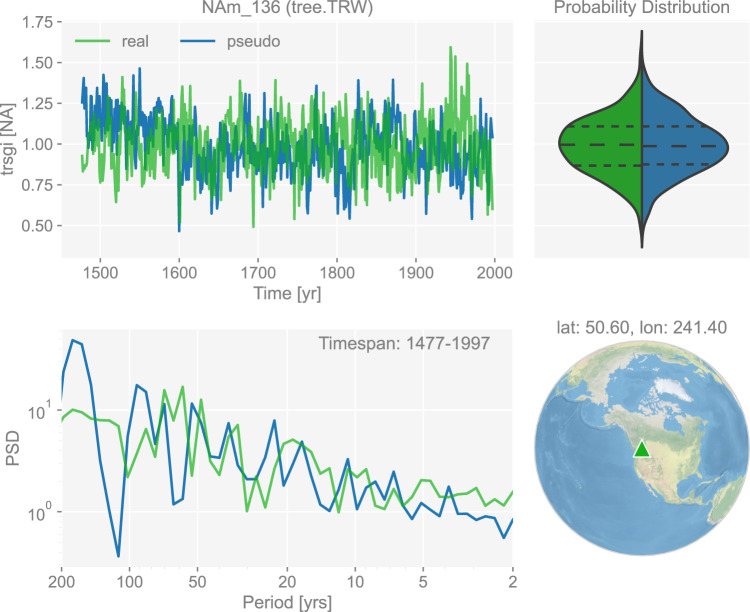
The dashboard for the tree ring width record “NAm_136” in dataset “ppwn_SNRinf_rta”. The unit “NA” stands for “not applicable” as the variable is a standardized index and thus unitless. “PSD” refers to power spectral density and is in the unit of power (squared unit of the proxy variable) per year.

The four threshold parameters *T*_1_, *T*_2_, *M*_1_, *M*_2_ are crucial to the behavior of the model. We calibrate them against the CRUTS monthly temperature and precipitation observations^90^ version 4.05, using the Bayesian inference method elaborated in ref. ^76^. This essentially generates optimal posterior probability distributions for each threshold parameter by updating the prior distributions over Monte-Carlo iterations, and yields the estimate of each parameter following maximum likelihood estimation (MLE). With the calibrated parameters, iCESM-simulated monthly temperature and precipitation signals can be translated to the corresponding pseudo-TRW series. An example of the generated pseudo-TRW chronology and its comparison to the real-world counterpart in time and frequency domains is shown in Fig. 3.

#### Forward modeling of maximum latewood density (MXD)

**Fig. 4:**
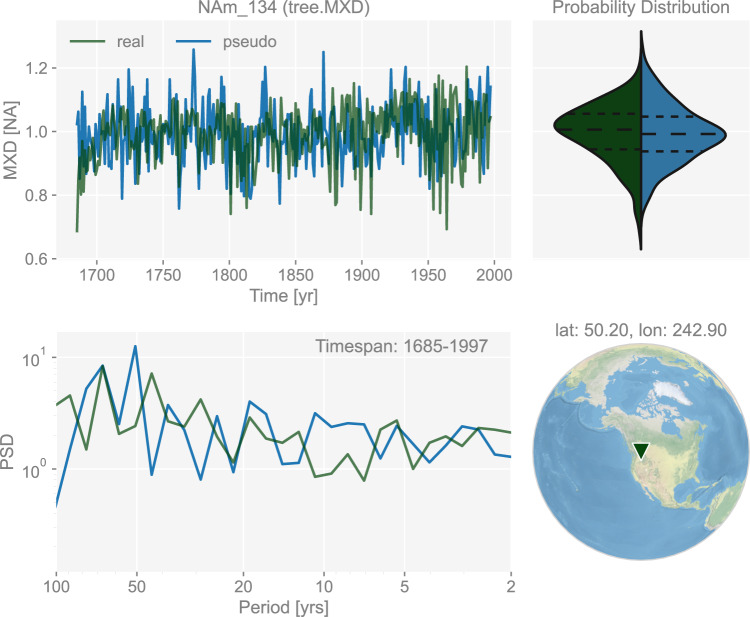
The dashboard for the maximum latewood density record “NAm_134” in dataset “ppwn_SNRinf_rta”. The unit “NA” stands for “not applicable” as the variable is a standardized index and thus unitless. “PSD” refers to power spectral density and is in the unit of power (squared unit of the proxy variable) per year.

Compared to TRW, maximum latewood density (MXD) more faithfully tracks growing season temperature history without distortions due to biological memory effects^49,50,68,69,84,91–95^. As there is not yet a published, tractable proxy system model for MXD, here we use a simple univariate linear model to emulate the behavior of MXD series:

4 $${\rm{MXD}}=a{T}_{{\rm{seasonal}}}+b,$$

where *a* represents a linear slope factor, *T*_seasonal_ the average temperature over the growing season, and *b* the intercept. The growing season is calibrated against the CRUTS dataset, version 4.05. Following ref. ^48^, the season that yields the optimal regression skill is picked from an expert-curated pool of growing season candidates, including the default calendar year option (Jan-Dec) and variants of warm seasons (i.e., Jun-Aug, Mar-Aug, Jun-Nov for Northern Hemisphere, and Dec-Feb, Dec-May, Sep-Feb for Southern Hemisphere) during which trees are expected to grow. An example of a generated pseudo-MXD chronology is shown in Fig. 4.

#### Forward modeling of coral and sclerosponge *δ*^18^O

**Fig. 5:**
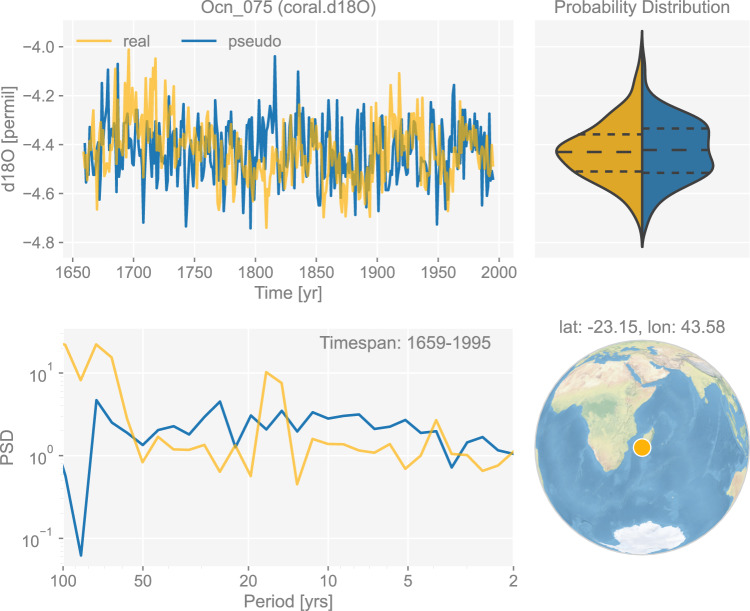
The dashboard for the coral *δ*^18^O record “Ocn_075” in dataset “ppwn_SNRinf_rta”. “PSD” refers to power spectral density and is in the unit of power (squared unit of the proxy variable) per year.

In contrast to trees, corals and sclerosponges mainly cover the tropical ocean regions and are thus of great importance to investigating tropical climate variability, including El Niño Southern Oscillation (ENSO)^17,96–101^. Following Brown *et al*.^102^, we use a bilinear model to simulate coral and sclerosponge *δ*^18^O based on the annual sea surface temperature (SST) and seawater *δ*^18^O (denoted as *δ*^18^O_sw_) signals:

5 $${\delta }^{18}{\rm{O}}=a{\rm{SST}}+b{\delta }^{18}{{\rm{O}}}_{{\rm{sw}}},$$

where *a* = −0.22 represents the linear slope factor, and *b* = 0.97002 the conversion factor from VSMOW to VPDB. Thompson *et al*.^103^ state that since the *δ*^18^O_sw_ network is scarce, they use sea surface salinity (SSS) to estimate *δ*^18^O_sw_. However, a salinity-based PSM is reliant on the SSS/*δ*^18^O_sw_ relationships that are known to be nonstationary and are based on extremely limited observational data^104^; the original formulation based on *δ*^18^O_sw_ is thus preferable given that the iCESM output is leveraged. An example of the generated pseudo-coral/sclerosponge *δ*^18^O chronology is shown in Fig. 5.

#### Forward modeling of coral and sclerosponge Sr/Ca

**Fig. 6:**
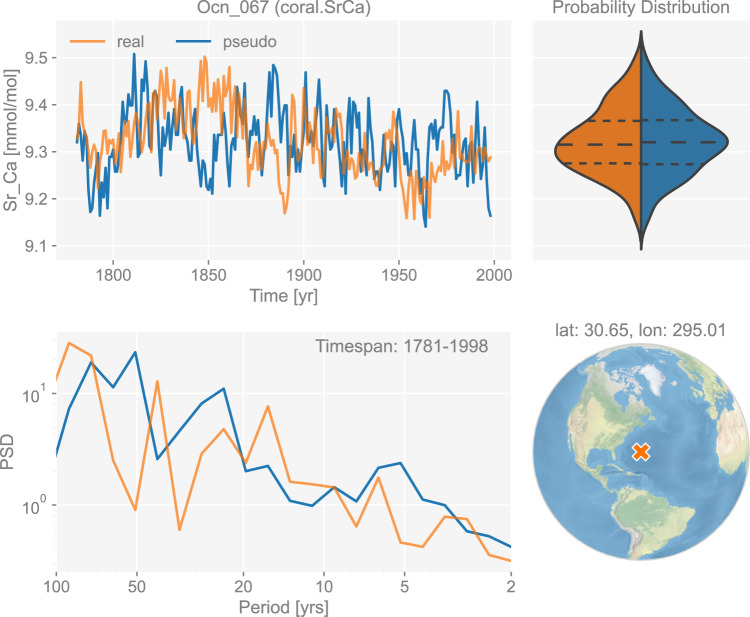
The dashboard for the coral Sr/Ca record “Ocn_067” in dataset “ppwn_SNRinf_rta”. “PSD” refers to power spectral density and is in the unit of power (squared unit of the proxy variable) per year.

The skeletal trace element ratio Sr/Ca in corals and sclerosponges has a straightforward temperature interpretation. Following Corrège *et al*.^105^, we apply a simple univariate linear model based on the annual SST signal, but with fixed parameters:

6 $${\rm{Sr}}/{\rm{Ca}}=a{\rm{SST}}+b,$$

where *a* represents the linear slope factor with a Gaussian distribution with mean of −0.06 and standard deviation of 0.01, and *b* is the intercept with a mean value around 10.553 based on Table 1 in Corrège *et al*.^105^. In this study, we take *a* = −0.06 and *b* = 10.553. An example of the generated pseudo-coral/sclerosponge Sr/Ca chronology is shown in Fig. 6.

**Table 1:** The seasonality of each lake varve thickness site.

PAGES2k Site ID	Seasonality
Arc_001	Jun - Aug
Arc_014	Dec - Mar
Arc_020	Jun - Aug
Arc_022	Jun - Aug
Arc_025	Jun - Aug
Arc_030	Jul - Sep
Arc_072	Mar - Oct
Arc_076	Jun

#### Forward modeling of ice core *δ*^18^O

Glacier ice cores mainly cover the polar and mountain regions, where trees cannot grow. They are usually well-preserved and span a long time interval with annual time resolution, and are important to investigate long-term climate change. For ice core *δ*^18^O, we apply the corresponding module in the PRYSM toolbox^38^, which is based on the work of Johnsen^106^, Whillans and Grootes^107^, Cuffey and Steig^108^, Johnsen *et al*.^109^, and Küttel *et al*.^110^.

Its sensor model takes precipitation-weighted *δ*^18^O to emulate the *δ*^18^O input to ice:

7 $${\delta }^{18}{O}_{{\rm{weighted}}}=\sum \left(p{\delta }^{18}{O}_{p}\right)/\sum p,$$

where *p* represents the monthly precipitation amount, and *δ*^18^*O*_*p*_ the precipitation *δ*^18^*O*. The precipitation-weighted *δ*^18^O is then corrected based on the elevation difference between the proxy site and its nearest model grid point with a rate of −0.25 per 100 meters. Next, its archive model emulates the compaction and diffusion processes of isotopes in ice via a convolution with a Gaussian kernel *G*:

8 $${\delta }^{18}{O}_{{\rm{ice}}}=G\ast {\delta }^{18}{O}_{{\rm{weighted}}\cdot }$$

**Fig. 7:**
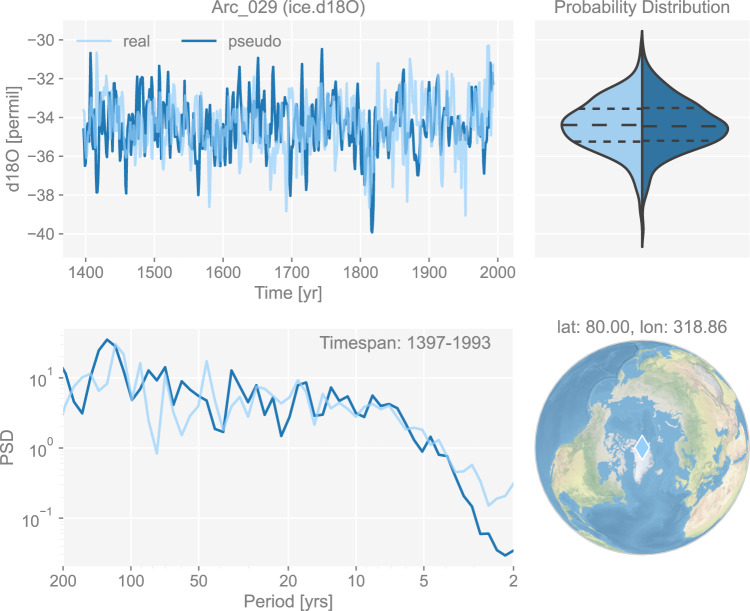
The dashboard for the ice core *δ*^18^O record “Arc_029” in dataset “ppwn_SNRinf_rta”. “PSD” refers to power spectral density and is in the unit of power (squared unit of the proxy variable) per year.

An example of the generated pseudo-ice core *δ*^18^O chronology is shown in Fig. 7.

#### Forward modeling of lake varve thickness

Varves, or annually laminated sediments, can be valuable temperature proxies for the Common Era due to their high-resolution and because they can be found in areas where other annually-resolved archives are absent. Varve thickness or mass accumulation rate are directly related to sediment input and deposition, which in turn can be strongly related to climate in some lakes, however many phenomena can affect varve thickness, and the relationship between climatic and environmental drivers and varve thickness is often complex and typically varies from lake to lake^111^. Temperature-driven varve thickness records are most common in the Arctic, where summer temperature can have strong and direct impacts on sediment transportation by melting winter snowpack and glaciers and extending the ice-free season.

The PAGES 2k Phase 2 database includes eight sites with varve thickness records interpreted to respond to temperature. Mechanistically simulating varve thickness is complex, highly site-specific, and not practical for most PPE studies. Nevertheless, most varve thickness records share characteristics that are readily and simply simulated. There are two key processes that we simulate. First, because varve thickness measures a depositional process, the distribution of varve thickness is zero-bounded and right-skewed, and is appropriately modeled with a Poisson or Gamma distribution^112^. Second, varve thickness records typically include substantial year-to-year memory. Unlike most sedimentary records, this is not due to bioturbation or post-depositional mixing (as this would destroy the laminations). However, glacial and sedimentary processes in the watershed and in the lake can be prone to significant memory, which affects the spectral characteristics of varve thickness records.

**Fig. 8:**
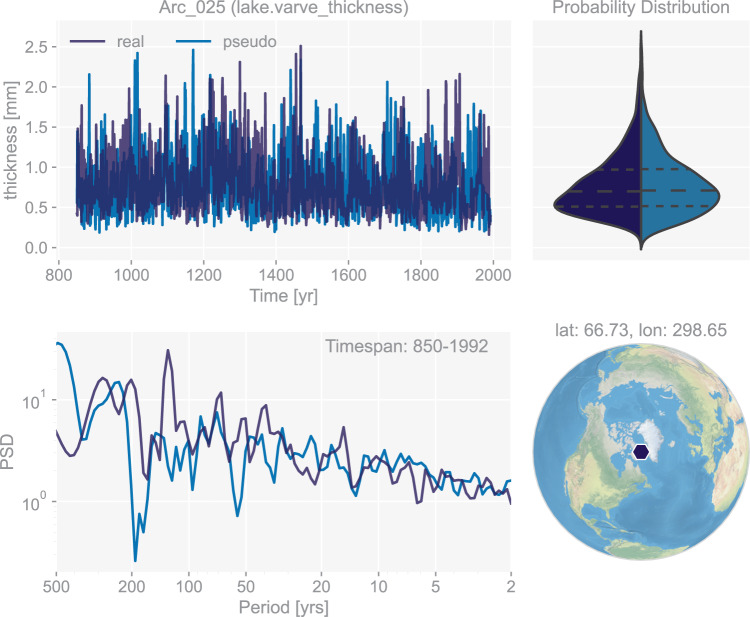
The dashboard for the lake varve thickness record “Arc_025” in dataset “ppwn_SNRinf_rta”. “PSD” refers to power spectral density and is in the unit of power (squared unit of the proxy variable) per year.

Here, we apply a simple model as below:

9 $${\rm{thickness}}=\Gamma \left({T}_{{\rm{seasonal}}}\right)+\Gamma (b),$$

where Γ(·) represents a mapping from the original distribution to a Gamma distribution, *T*_seasonal_ is the seasonally-averaged temperature calculated based on the seasonality metadata of each site (Table 1)^43^, and *b* is a realization of fractional Brownian motion with Hurst index *H* = 0.75 and SNR = 1, a combination we find fits well with the real records. An example of the generated pseudo-lake varve thickness chronology is shown in Fig. 8.

### Pseudoproxy production workflow

**Fig. 9:**
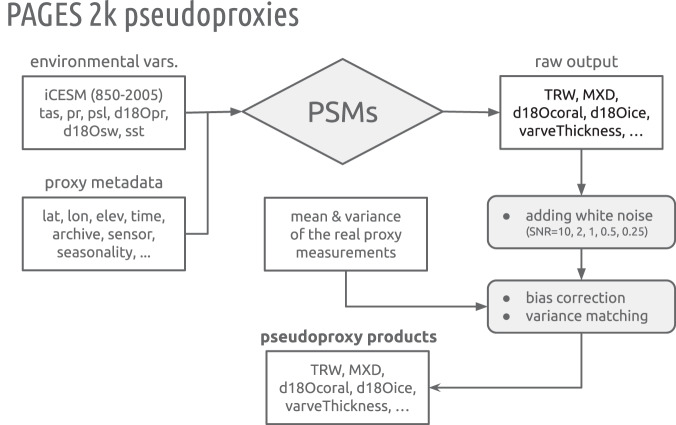
Flow chart of the general procedure for pseudoproxy generation.

Figure 9 shows the general procedure for pseudoproxy generation. The starting point is the isotope-enabled Community Earth System Model (iCESM) last millennium plus historical simulation^52^ (Section *Reference Climate*), chosen so that the isotope-related proxies can be simulated with minimal assumptions.

Environmental variables are taken from the iCESM output, including air surface temperature, precipitation rate, sea level pressure, precipitation *δ*^18^O, seawater *δ*^18^O, and sea surface temperature (SST). Proxy metadata are taken from the PAGES 2k dataset (Section *Proxy Network*), including the location information, time axis, archive type, sensor, species, seasonality, etc.

These two sources of information (environmental variables and proxy metadata) are then fed to the PSMs for tree-ring width (TRW), maximum latewood density (MXD), coral/sclerosponge *δ*^18^O, coral/sclerosponge Sr/Ca, lake varve thickness, and ice core *δ*^18^O, which translate the climatic signals and generate the raw output in proxy space (Section *Proxy Modeling*).

The raw output is then treated as signal, and white noise is added with a set of signal-to-noise ratio (SNR, defined in standard deviation^24,26,89^) options (∞, 10, 2, 1, 0.5, 0.25)^22,28^, where SNR = ∞ is a noise-free case, SNR = 1 means that the signal and noise share an equal standard deviation, etc. We generate datasets with two types of temporal sampling: (1) full annual sampling over 850–2005 CE, and (2) the realistic temporal availability of each record (Fig. 2).

Because iCESM is a biased representation of reality, the pseudoproxies generated by this workflow inherit some of the same biases in low-order statistics like mean and variance. To facilitate comparison with real-world records, we apply a bias correction and variance matching against the real records, according to the mean and variance of the real proxy measurements over the common timespan to the pseudoproxy counterpart. Note that this step shifts and scales the time series, but has no impact on the statistical distribution (e.g., Gaussian or Gamma), nor the spectral characteristics (i.e., scaling slopes and peaks) of the simulated proxies.

As a benchmark, we also generate pseudoproxies following the traditional temperature-plus-noise method: the temperature signal at the grid cell nearest each proxy site is added with white noise with the same set of SNR options and the same two types of temporal sampling.

This workflow results in multiple pseudoproxy emulations of the PAGES 2k network, differing in:

**design** either “temperature-plus-white-noise” model (tpwn) or using the pseudoproxy models described above, with added white noise (ppwn)

**noise level** as quantified by the SNR of ∞ (pure signal), 10, 2, 1, 0.5 or 0.25.

**temporal sampling** either full annual sampling over 850–2005 CE (fta), or the realistic temporal availability of Fig. 2 (rta).

## Data Records

Table 2 lists the pseudoproxy datasets generated in this study, which we call “pseudoPAGES2k”. The dataset IDs indicate the property of each dataset. For instance, “tpwn_SNR10_fta” means that the pseudoproxies are generated with the temperature-plus-white-noise method with SNR equals to 10 and full temporal availability, while “ppwn_SNR0.5_rta” means that the pseudoproxies are generated via the PSM hierarchy with white noise added on and SNR equals to 0.5, and the realistic temporal availability is applied, and so on and so forth. The datasets are archived at Zenodo^113^ (10.5281/zenodo.7652533).

**Table 2:** A list of the “pseudoPAGES2k” pseudoproxy datasets.

Dataset ID	Signal	Noise	SNR	Sampling
ppwn_SNRinf_rta	PSM generated	none	inf	realistic
ppwn_SNR10_rta	PSM generated	white noise	10	realistic
ppwn_SNR2_rta	PSM generated	white noise	2	realistic
ppwn_SNR1_rta	PSM generated	white noise	1	realistic
ppwn_SNR0.5_rta	PSM generated	white noise	0.5	realistic
ppwn_SNR0.25_rta	PSM generated	white noise	0.25	realistic
ppwn_SNRinf_fta	PSM generated	none	inf	full
ppwn_SNR10_fta	PSM generated	white noise	10	full
ppwn_SNR2_fta	PSM generated	white noise	2	full
ppwn_SNR1_fta	PSM generated	white noise	1	full
ppwn_SNR0.5_fta	PSM generated	white noise	0.5	full
ppwn_SNR0.25_fta	PSM generated	white noise	0.25	full
tpwn_SNR10_rta	nearest temperature	white noise	10	realistic
tpwn_SNR2_rta	nearest temperature	white noise	2	realistic
tpwn_SNR1_rta	nearest temperature	white noise	1	realistic
tpwn_SNR0.5_rta	nearest temperature	white noise	0.5	realistic
tpwn_SNR0.25_rta	nearest temperature	white noise	0.25	realistic
tpwn_SNR10_fta	nearest temperature	white noise	10	full
tpwn_SNR2_fta	nearest temperature	white noise	2	full
tpwn_SNR1_fta	nearest temperature	white noise	1	full
tpwn_SNR0.5_fta	nearest temperature	white noise	0.5	full
tpwn_SNR0.25_fta	nearest temperature	white noise	0.25	full

“SNR” refers to signal-to-noise ratio defined in standard deviation^24,26,89^. The full sampling refers to 850–2005 CE with annual resolution, and the realistic sampling refers to the realistic temporal availability of each record.

The “iCESM1” last millennium simulation (iCESM-LM) used in this study can be accessed at a data server hosted by Rorbert Tardif at University of Washington (https://atmos.washington.edu/~rtardif/LMR/prior).

The PAGES 2k Phase 2 Network used in this study can be accessed at the National Center for Environmental Information’s World Data Service for Paleoclimatology (https://www.ncei.noaa.gov/access/paleo-search/study/21171).

## Technical Validation

To verify if the generation procedure (Fig. 9) yields a realistic pseudoproxy emulation of the PAGES 2k database, we validate the generated pseudoproxies against the original records’ statistics, in both time and frequency domains. We emphasize that this is a validation specifically of the realism (and therefore utility) of the pseudoproxy generation procedure, rather than an evaluation of any single PSM or GCM, which has been done elsewhere. Rather, we aim to show that, coupling these models–imperfect though they may be–can produce pseudoproxies that emulate key characteristics of the target series. In the time domain, a good pseudoproxy emulation should reproduce the probability distribution shape of the real proxies; this may be assessed via split violin plots. In the frequency domain, a good emulation should reproduce the power spectral density (PSD) of the target series, indicating the energy partitioning per frequency interval, particularly the periodic and continuum^114^ characteristics of the series.

**Fig. 10:**
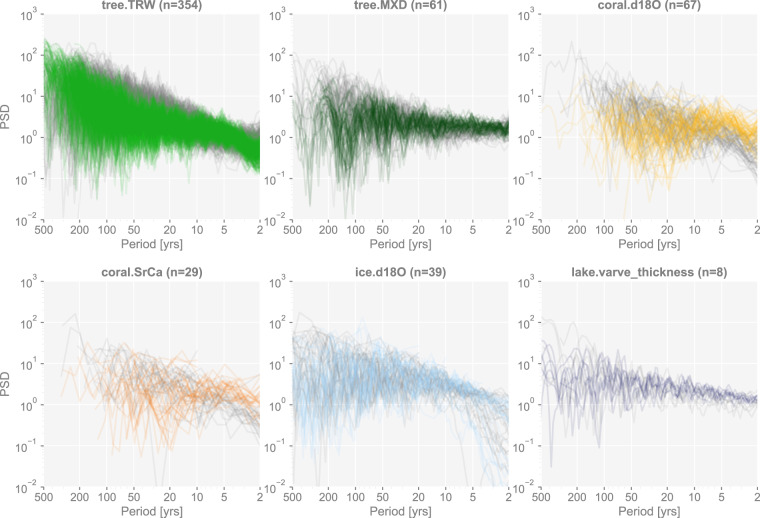
Spectral analysis of the pseudoproxy records in dataset “ppwn_SNRinf_rta” by proxy type. The gray curves denote the power spectral density (PSD, in the unit of power per year, i.e., squared unit of the proxy variable per year) of the real records, while the colored curves denote that of the pseudoproxy records.

Figures 3 to 8 show examples for specific records, one site per proxy type. Since the real records may be unevenly-spaced in time, we leverage the Weighted Wavelet Z-transform (WWZ) method implemented in Pyleoclim^115^, to obtain the PSD curves. As illustrated by the PSD plots and the probability distribution plots, the pseudoproxies show an overall good agreement with the real records, including, for instance, the steep attenuation of high-frequencies in the ice core *δ*^18^O record shown in Fig. 7, and the long tail distribution of the varve thickness record shown in Fig. 8. To validate thoroughly the spectral characteristics, Fig. 10 shows the spectral analysis by proxy types. It can be seen that overall good agreement is achieved between the pseudoproxies and their real counterparts, indicating a realistic emulation from the spectral perspective. This should result in more realistic assessments of the spectral characteristics of reconstruction skill. We emphasize that the procedure of bias correction and variance matching has no bearing on these aspects of the validation, as it simply adds a scale and offset to the pseudoproxies, without modifying their probability distribution shape or spectral characteristics.

## Usage Notes

To illustrate the many potential uses of this dataset, we provide Jupyter notebooks (*Code Availability*) for the basic analysis and visualization of the dataset, as well as applications to climate field reconstruction. Specifically, we provide Python-based examples of:

- Loading and visualizing the pseudoPAGES2k dataset.
- Filtering the pseudoPAGES2k dataset according to various criteria.
- Generating dashboards like Figs. 3–8.
- How to apply Paleoclimate Data Assimilation (in the vein of the Last Millennium Reanalysis^48^) to the pseudoPAGES2k dataset, and its use for benchmarking climate field reconstruction methods.

Other potential uses of this dataset and its production workflow include optimal sampling design^116^. A natural extension would be to add age uncertainties to these pseudoproxies, as done in ref. ^31^.

## Data Availability

The Jupyter notebooks illustrating the usage of the pseudoPAGES2k dataset can be accessed at 10.5281/zenodo.7652533 or https://github.com/fzhu2e/paper-pseudoPAGES2k.

## References

[1] IPCC. Summary for policymakers. In Masson-Delmotte, V. *et al*. (eds.) *Climate Change 2021: The Physical Science Basis. Contribution of Working Group I to the Sixth Assessment Report of the Intergovernmental Panel on Climate Change* (Cambridge University Press, 2021).

[2] Tingley MP (2012). Piecing together the past: statistical insights into paleoclimatic reconstructions. Quaternary Science Reviews.

[3] Jones JM, Widmann M (2004). Early peak in Antarctic oscillation index. Nature.

[4] Goosse H (2006). The origin of the European “Medieval Warm Period”. Climate of the Past.

[5] Gebhardt C, Kühl N, Hense A, Litt T (2008). Reconstruction of Quaternary temperature fields by dynamically consistent smoothing. Climate Dynamics.

[6] Widmann M, Goosse H, van der Schrier G, Schnur R, Barkmeijer J (2010). Using data assimilation to study extratropical Northern Hemisphere climate over the last millennium. Climate of the Past.

[7] Goosse, H. *et al*. Reconstructing surface temperature changes over the past 600 years using climate model simulations with data assimilation. Journal of Geophysical Research: Atmospheres **115**, 10.1029/2009JD012737 (2010).

[8] Annan JD, Hargreaves JC (2013). A new global reconstruction of temperature changes at the Last Glacial Maximum. Climate of the Past.

[9] Steiger NJ, Hakim GJ, Steig EJ, Battisti DS, Roe GH (2014). Assimilation of Time-Averaged Pseudoproxies for Climate Reconstruction. Journal of Climate.

[10] Hakim GJ (2016). The last millennium climate reanalysis project: Framework and first results. Journal of Geophysical Research: Atmospheres.

[11] Franke J, Brönnimann S, Bhend J, Brugnara Y (2017). A monthly global paleo-reanalysis of the atmosphere from 1600 to 2005 for studying past climatic variations. Scientific Data.

[12] Acevedo W, Fallah B, Reich S, Cubasch U (2017). Assimilation of pseudo-tree-ring-width observations into an atmospheric general circulation model. Climate of the Past.

[13] Steiger NJ, Smerdon JE, Cook ER, Cook BI (2018). A reconstruction of global hydroclimate and dynamical variables over the Common Era. Scientific Data.

[14] Tierney JE (2020). Glacial cooling and climate sensitivity revisited. Nature.

[15] Osman MB (2021). Globally resolved surface temperatures since the Last Glacial Maximum. Nature.

[16] King JM (2021). A data assimilation approach to last millennium temperature field reconstruction using a limited high-sensitivity proxy network. Journal of Climate.

[17] Zhu F (2022). A re-appraisal of the ENSO response to volcanism with paleoclimate data assimilation. Nature Communications.

[18] Shoji S, Okazaki A, Yoshimura K (2022). Impact of proxies and prior estimates on data assimilation using isotope ratios for the climate reconstruction of the last millennium. Earth and Space Science.

[19] Valler V, Franke J, Brugnara Y, Brönnimann S (2022). An updated global atmospheric paleo-reanalysis covering the last 400 years. Geoscience Data Journal.

[20] Annan JD, Hargreaves JC, Mauritsen T (2022). A new global surface temperature reconstruction for the Last Glacial Maximum. Climate of the Past.

[21] Smerdon JE, Pollack HN (2016). Reconstructing earth’s surface temperature over the past 2000 years: the science behind the headlines. Wiley Interdisciplinary Reviews: Climate Change n/a–n/a.

[22] Wang J, Emile-Geay J, Guillot D, McKay NP, Rajaratnam B (2015). Fragility of reconstructed temperature patterns over the common era: Implications for model evaluation. Geophysical Research Letters.

[23] Neukom R, Steiger N, Gómez-Navarro JJ, Wang J, Werner JP (2019). No evidence for globally coherent warm and cold periods over the preindustrial common era. Nature.

[24] Smerdon JE (2012). Climate models as a test bed for climate reconstruction methods: pseudoproxy experiments. Wiley Interdisciplinary Reviews: Climate Change.

[25] Smerdon JE, Kaplan A, Chang D, Evans MN (2010). A Pseudoproxy Evaluation of the CCA and RegEM Methods for Reconstructing Climate Fields of the Last Millennium. Journal of Climate.

[26] Smerdon, J. E., Kaplan, A., Zorita, E., González–Rouco, J. F. & Evans, M. N. Spatial performance of four climate field reconstruction methods targeting the Common Era. Geophysical Research Letters **38**, 10.1029/2011GL047372 (2011).

[27] Smerdon JE, Kaplan A, Amrhein DE (2010). Erroneous model field representations in multiple pseudoproxy studies: Corrections and implications*. J. Clim..

[28] Wang J, Emile-Geay J, Guillot D, Smerdon JE, Rajaratnam B (2014). Evaluating climate field reconstruction techniques using improved emulations of real-world conditions. Climate of the Past.

[29] Evans MN, Smerdon JE, Kaplan A, Tolwinski–Ward SE, González–Rouco JF (2014). Climate field reconstruction uncertainty arising from multivariate and nonlinear properties of predictors. Geophysical Research Letters.

[30] Smerdon, J. E., Coats, S. & Ault, T. R. Model-dependent spatial skill in pseudoproxy experiments testing climate field reconstruction methods for the Common Era. Climate Dynamics 1–22, 10.1007/s00382-015-2684-0 (2015).

[31] Nilsen T, Talento S, Werner JP (2021). Constraining two climate field reconstruction methodologies over the north atlantic realm using pseudo-proxy experiments. Quaternary Science Reviews.

[32] Mann ME, Rutherford S (2002). Climate reconstruction using ‘Pseudoproxies’. Geophys. Res. Lett..

[33] Rutherford S, Mann ME, Delworth TL, Stouffer RJ (2003). Climate field reconstruction under stationary and nonstationary forcing. J. Clim..

[34] Rutherford S (2005). Proxy-Based Northern Hemisphere Surface Temperature Reconstructions: Sensitivity to Method, Predictor. Network, Target Season, and Target Domain. J. Clim..

[35] Mann ME, Rutherford S, Wahl E, Ammann C (2007). Robustness of proxy-based climate field reconstruction methods. Journal of Geophysical Research (Atmospheres).

[36] Gómez-Navarro JJ, Zorita E, Raible CC, Neukom R (2017). Pseudo-proxy tests of the analogue method to reconstruct spatially resolved global temperature during the Common Era. Climate of the Past.

[37] Evans MN, Tolwinski-Ward SE, Thompson DM, Anchukaitis KJ (2013). Applications of proxy system modeling in high resolution paleoclimatology. Quaternary Science Reviews.

[38] Dee S (2015). PRYSM: An open-source framework for PRoxY system modeling, with applications to oxygen-isotope systems. J. Adv. Model. Earth Syst..

[39] Dee SG, Steiger NJ, Emile-Geay J, Hakim GJ (2016). On the utility of proxy system models for estimating climate states over the common era. Journal of Advances in Modeling Earth Systems.

[40] Dee SG (2017). Improved spectral comparisons of paleoclimate models and observations via proxy system modeling: Implications for multi-decadal variability. Earth and Planetary Science Letters.

[41] Dee SG, Russell JM, Morrill C, Chen Z, Neary A (2018). PRYSM v2.0: A Proxy System Model for Lacustrine Archives. Paleoceanography and Paleoclimatology.

[42] Bothe O, Wagner S, Zorita E (2019). Simple noise estimates and pseudoproxies for the last 21000 years. Earth System Science Data.

[43] PAGES2k Consortium (2017). A global multiproxy database for temperature reconstructions of the Common Era. Scientific Data.

[44] Widmann, M., Franke, J., Goosse, H., Hakim, G. & Steiger, N. The DAPS data assimilation intercomparison experiment. In EGU General Assembly Conference Abstracts, EGU General Assembly Conference Abstracts, 19100 (2018).

[45] Neukom R (2019). Consistent multidecadal variability in global temperature reconstructions and simulations over the common era. Nature Geoscience.

[46] Wang J (2020). Evaluation of multidecadal and longer-term temperature changes since 850 CE based on Northern Hemisphere proxy-based reconstructions and model simulations. Science China Earth Sciences.

[47] St. Klippel L, George S, Büntgen U, Krusic PJ, Esper J (2020). Differing pre-industrial cooling trends between tree rings and lower-resolution temperature proxies. Climate of the Past.

[48] Tardif R (2019). Last Millennium Reanalysis with an expanded proxy database and seasonal proxy modeling. Climate of the Past.

[49] Zhu F, Emile-Geay J, Hakim GJ, King J, Anchukaitis KJ (2020). Resolving the Differences in the Simulated and Reconstructed Temperature Response to Volcanism. Geophysical Research Letters.

[50] Anchukaitis KJ, Smerdon JE (2022). Progress and uncertainties in global and hemispheric temperature reconstructions of the common era. Quaternary Science Reviews.

[51] Guillot D, Rajaratnam B, Emile-Geay J (2015). Statistical paleoclimate reconstructions via Markov random fields. The Annals of Applied Statistics.

[52] Brady E (2019). The Connected Isotopic Water Cycle in the Community Earth System Model Version 1. Journal of Advances in Modeling Earth Systems.

[53] Nusbaumer J, Wong TE, Bardeen C, Noone D (2017). Evaluating hydrological processes in the Community Atmosphere Model Version 5 (CAM5) using stable isotope ratios of water. Journal of Advances in Modeling Earth Systems.

[54] Wong TE, Nusbaumer J, Noone DC (2017). Evaluation of modeled land-atmosphere exchanges with a comprehensive water isotope fractionation scheme in version 4 of the Community Land Model. Journal of Advances in Modeling Earth Systems.

[55] Oleson, K. W. *et al*. Technical description of version 4.0 of the Community Land Model (CLM). Tech. Rep., National Center for Atmospheric Research (2010).

[56] Zhang J (2017). Asynchronous warming and *δ*18O evolution of deep Atlantic water masses during the last deglaciation. Proceedings of the National Academy of Sciences.

[57] Otto-Bliesner BL (2015). Climate variability and change since 850 ce: An ensemble approach with the community earth system model. Bulletin of the American Meteorological Society.

[58] Vieira LEA, Solanki SK, Krivova NA, Usoskin I (2011). Evolution of the solar irradiance during the Holocene. Astronomy & Astrophysics.

[59] Schmidt GA (2012). Climate forcing reconstructions for use in PMIP simulations of the Last Millennium (v1.1). Geoscientific Model Development.

[60] Gao, C., Robock, A. & Ammann, C. Volcanic forcing of climate over the past 1500 years: An improved ice core-based index for climate models. Journal of Geophysical Research: Atmospheres **113**, 10.1029/2008JD010239 (2008).

[61] Ammann, C. M., Meehl, G. A., Washington, W. M. & Zender, C. S. A monthly and latitudinally varying volcanic forcing dataset in simulations of 20th century climate. Geophysical Research Letters **30**, 10.1029/2003GL016875 (2003).

[62] Pongratz, J., Reick, C., Raddatz, T. & Claussen, M. A reconstruction of global agricultural areas and land cover for the last millennium. Global Biogeochemical Cycles **22**, 10.1029/2007GB003153 (2008).

[63] Hurtt GC (2011). Harmonization of land-use scenarios for the period 1500– 2100: 600 years of global gridded annual land-use transitions, wood harvest, and resulting secondary lands. Climatic Change.

[64] Berger A, Loutre M-F, Tricot C (1993). Insolation and Earth’s orbital periods. Journal of Geophysical Research: Atmospheres.

[65] Zhu F (2023). Zenodo.

[66] Fritts HC (1966). Growth-rings of trees: their correlation with climate. Science.

[67] Krakauer, N. Y. & Randerson, J. T. Do volcanic eruptions enhance or diminish net primary production? Evidence from tree rings. Global Biogeochemical Cycles **17**, 10.1029/2003GB002076 (2003).

[68] Frank D, Büntgen U, Böhm R, Maugeri M, Esper J (2007). Warmer early instrumental measurements versus colder reconstructed temperatures: shooting at a moving target. Quaternary Science Reviews.

[69] Esper J, Schneider L, Smerdon JE, Schöne BR, Büntgen U (2015). Signals and memory in tree-ring width and density data. Dendrochronologia.

[70] Stoffel M (2015). Estimates of volcanic-induced cooling in the Northern Hemisphere over the past 1,500 years. Nature Geoscience.

[71] Zhang H (2015). Modified climate with long term memory in tree ring proxies. Environmental Research Letters.

[72] Lücke L, Hegerl G, Schurer A, Wilson R (2019). Effects of memory biases on variability of temperature reconstructions. Journal of Climate.

[73] Fritts, H. *et al*. User manual for treering 2000. Tech. Rep., Laboratory of Tree-Ring Research (2000).

[74] Vaganov, E. A., Hughes, M. K. & Shashkin, A. V. Growth Dynamics of Conifer Tree Rings: Images of Past and Future Environments. Ecological Studies (Springer-Verlag, Berlin Heidelberg, 2006).

[75] Tolwinski-Ward SE, Evans MN, Hughes MK, Anchukaitis KJ (2011). An efficient forward model of the climate controls on interannual variation in tree-ring width. Climate Dynamics.

[76] Tolwinski-Ward SE, Anchukaitis KJ, Evans MN (2013). Bayesian parameter estimation and interpretation for an intermediate model of tree-ring width. Climate of the Past.

[77] Tolwinski-Ward SE, Tingley MP, Evans MN, Hughes MK, Nychka DW (2015). Probabilistic reconstructions of local temperature and soil moisture from tree-ring data with potentially time-varying climatic response. Climate Dynamics.

[78] Rezsöhazy, J. *et al*. Application and evaluation of the dendroclimatic process-based model MAIDEN during the last century in Canada and Europe. Climate of the Past **16**, 1043–1059, 10.5194/cp-16-1043-2020. Publisher: Copernicus GmbH (2020).

[79] RezsÃ¶hazy J, Gennaretti F, Goosse H, Guiot J (2021). Testing the performance of dendroclimatic process-based models at global scale with the PAGES2k tree-ring width database. Climate Dynamics.

[80] Druel A, Ciais P, Krinner G, Peylin P (2019). Modeling the Vegetation Dynamics of Northern Shrubs and Mosses in the ORCHIDEE Land Surface Model. Journal of Advances in Modeling Earth Systems.

[81] Evans, M. N., Zhu, F., Rezsöhazy, J. & Jeong, J. What complexity PSM for paleoclimate data assimilation? Results from a tree-ring width data model intercomparison study. In EOS, Transactions, AGU. Abstract PP041-0011 presented at the AGU Fall 2020 Meeting, 15 Dec (2020).

[82] Franke J, Frank D, Raible CC, Esper J, Brönnimann S (2013). Spectral biases in tree-ring climate proxies. Nature Climate Change.

[83] Zhang H (2015). Modified climate with long term memory in tree ring proxies. Environmental Research Letters.

[84] Esper J (2018). Large-scale, millennial-length temperature reconstructions from tree-rings. Dendrochronologia.

[85] Büntgen U (2021). The influence of decision-making in tree ring-based climate reconstructions. Nature Communications.

[86] Kirchner JW (2005). Aliasing in 1/f(alpha) noise spectra: origins, consequences, and remedies. Physical Review. E, Statistical, Nonlinear, and Soft Matter Physics.

[87] Huybers P, Curry W (2006). Links between annual, Milankovitch and continuum temperature variability. Nature.

[88] Zhu, F. *et al*. Climate models can correctly simulate the continuum of global-average temperature variability. Proceedings of the National Academy of Sciences 201809959, 10.1073/pnas.1809959116 (2019). 10.1073/pnas.1809959116 PMC6500133 30988176

[89] Mann, M. E., Rutherford, S., Wahl, E. & Ammann, C. Robustness of proxy-based climate field reconstruction methods. Journal of Geophysical Research: Atmospheres **112**, 10.1029/2006JD008272 (2007).

[90] Harris I, Jones PD, Osborn TJ, Lister DH (2014). Updated high-resolution grids of monthly climatic observations – the CRU TS3.10 Dataset. International Journal of Climatology.

[91] D’Arrigo R, Wilson R, Anchukaitis KJ (2013). Volcanic cooling signal in tree ring temperature records for the past millennium. Journal of Geophysical Research: Atmospheres.

[92] Schneider L (2015). Revising midlatitude summer temperatures back to ad 600 based on a wood density network. Geophysical Research Letters.

[93] Wilson R (2016). Last millennium northern hemisphere summer temperatures from tree rings: Part i: The long term context. Quaternary Science Reviews.

[94] Anchukaitis KJ (2017). Last millennium northern hemisphere summer temperatures from tree rings: Part ii, spatially resolved reconstructions. Quaternary Science Reviews.

[95] Björklund J (2019). Scientific merits and analytical challenges of tree-ring densitometry. Reviews of Geophysics.

[96] Cobb KM, Charles CD, Cheng H, Edwards RL (2003). El Niño/Southern Oscillation and tropical Pacific climate during the last millennium. Nature.

[97] Cobb KM (2013). Highly Variable El Niño–Southern Oscillation Throughout the Holocene. Science.

[98] Emile-Geay J, Cobb KM, Mann ME, Wittenberg AT (2013). Estimating Central Equatorial Pacific SST Variability over the Past Millennium. Part I: Methodology and Validation. Journal of Climate.

[99] Emile-Geay J, Cobb KM, Mann ME, Wittenberg AT (2013). Estimating Central Equatorial Pacific SST Variability over the Past Millennium. Part II: Reconstructions and Implications. Journal of Climate.

[100] Tierney JE (2015). Tropical sea surface temperatures for the past four centuries reconstructed from coral archives. Paleoceanography.

[101] Emile-Geay, J., Cobb, K. M., Cole, J. E., Elliot, M. & Zhu, F. *Past ENSO Variability, chap*. **5**, 87–118, https://agupubs.onlinelibrary.wiley.com/doi/pdf/10.1002/496 9781119548164.ch5 (American Geophysical Union (AGU), 2020).

[102] Brown, J., Simmonds, I. & Noone, D. Modeling *δ*18 O in tropical precipitation and the surface ocean for present-day climate. Journal of Geophysical Research: Atmospheres **111**, 10.1029/2004JD005611 (2006).

[103] Thompson, D. M., Ault, T. R., Evans, M. N., Cole, J. E. & Emile-Geay, J. Comparison of observed and simulated tropical climate trends using a forward model of coral *δ* 18 O. Geophysical Research Letters **38**, 10.1029/2011GL048224 (2011).

[104] Stevenson S (2018). Twentieth Century Seawater *δ*18 O Dynamics and Implications for Coral-Based Climate Reconstruction. Paleoceanography and Paleoclimatology.

[105] Corrège T (2006). Sea surface temperature and salinity reconstruction from coral geochemical tracers. Palaeogeography, Palaeoclimatology, Palaeoecology.

[106] Johnsen S (1977). Stable isotope homogenization of polar firn and ice. Proceedings of the Symposium on Isotopes and Impurities in Snow and Ice.

[107] Whillans IM, Grootes PM (1985). Isotopic diffusion in cold snow and firn. Journal of Geophysical Research: Atmospheres.

[108] Cuffey KM, Steig EJ (1998). Isotopic diffusion in polar firn: implications for interpretation of seasonal climate parameters in ice-core records, with emphasis on central Greenland. Journal of Glaciology.

[109] Johnsen S (2000). Diffusion of stable isotopes in polar firn and ice. Proceedings of the Symposium on Isotopes and Impurities in Snow and Ice.

[110] Küttel M, Steig EJ, Ding Q, Monaghan AJ, Battisti DS (2012). Seasonal climate information preserved in West Antarctic ice core water isotopes: Relationships to temperature, large-scale circulation, and sea ice. Climate Dynamics.

[111] Hodder K, Gilbert R, Desloges J (2007). Glaciolacustrine varved sediment as an alpine hydroclimatic proxy. Journal of Paleolimnology.

[112] Blaauw, M. & Christen, J. Flexible paleoclimate age-depth models using an autoregressive gamma process. Bayesian Analysis **6**, 10.1214/ba/1339616472 (2011).

[113] Zhu F (2023). Zenodo.

[114] Ghil M (2002). Advanced spectral methods for climatic time series. Rev. Geophys..

[115] Khider D (2022). Pyleoclim: Paleoclimate Timeseries Analysis and Visualization With Python. Paleoceanography and Paleoclimatology.

[116] Comboul M, Emile-Geay J, Hakim GJ, Evans MN (2015). Paleoclimate sampling as a sensor placement problem. Journal of Climate.

